# Current News

**Published:** 1897-08

**Authors:** 


### Current News.

REPORT OF THE COMMITTEE ON IRREGULARITY AT THE PENNSYLVANIA STATE DENTAL SOCIETY, GLEN SUMMIT, PA., JULY 6, 1897.

To the Pennsylvania State Dental Society:

Gentlemen,—Your committee to whom was referred the question of irregularity committed at the session of this body, July, 1896, in the election of candidates for the Board of Examiners, would beg leave to report.

That it has given the subject careful consideration through several lengthy sessions, and has quite thoroughly examined all parties in interest, said examinations having been correctly reported stenographically and preserved as evidence of faithful work.

The testimony generally exhibited agreement upon the main facts, and where any discrepancy existed it seemed to be upon possible misconception of meanings.

It is the unanimous conclusion of the committee that the president at that meeting did make an informal expression of opinion regarding the disposition of surplus names upon the ballots, but they have reason to conclude, from the evidence, that this was given in the confusion incident to a general meeting and without any conception that it would be applied to the election of the Board of Examiners. On the other hand, it appears equally clear that two of the tellers, Drs. Roberts and Deane, construed this to apply universally throughout the balloting. Your committee are therefore unanimously of the opinion that the unfortunate occurrence was the result of a misunderstanding, combined with a lack of knowledge on the part of Dr. Deane, of the usage governing the reading of ballots, and they further failed to find that there was any evidence of fraud contemplated or attempted. They are further satisfied also that there was no evidence to influence a belief in a supposed conspiracy to defeat a candidate.

In view of these general statements, and in accordance with its duty, the committee would append the following resolutions for the consideration and adoption of the Pennsylvania State Dental Society:

*Resolved*, That after careful examination of the entire subject of irregularity said to have been committed, as heretofore stated, there has not been found the slightest evidence upon which to found a criminal charge.

*Resolved*, That this society extend to Dr. Deane the regrets of the entire body that he should have been subjected to unfounded and unwarranted suspicions during the past twelve months, but, while expressing this, it is important that it should be clearly understood that this difficulty might have been avoided by a better comprehension of the duties intrusted to the office of teller.

*Resolved*, That the committee, having performed all the duties required of it, is therefore discharged from further consideration of the subject.

(Signed) LOUIS JACK.
HENRY GERHART.
EDWIN T. DARBY.
G. W. KLUMP.
JAMES TRUMAN, *Chairman.*

The following preamble and resolution were offered by Dr. Truman, chairman of the committee, and ordered by the Society to be made part of the original report.

WHEREAS, The names of Drs. Robert Huey and H. H. Burchard, joint tellers with Dr. Deane in the counting of the ballots, having been brought unpleasantly into this matter, it may be inferred by those not conversant with all the facts, that these two were implicated in the charge of irregularity, therefore be it

*Resolved*, That neither in this Society nor in the dental profession throughout the State of Pennsylvania has there, at any time, been the slightest suspicion concerning their integrity or that they were cognizant of the irregularity, and this Society, without any reservations, relieves them of all responsibility in the matter.

---

### A VOLUNTARY STATEMENT BY DR. JOSEPH HEAD, WHICH WAS ACCEPTED BY THE PENNSYLVANIA STATE DENTAL SOCIETY, JULY, 1897.

INASMUCH as I sincerely regret that the issuing of the pamphlet reporting the action of the Odontological Society should have caused unjust suspicion to fall upon any one, and inasmuch as reprints of such action, which I, in accordance with my duty as editor, furnished to a member upon request, have been mailed, without the knowledge of the Odontological Society, to many of our profession, causing, by their incomplete evidence, grave injustice to three respected gentlemen, it will be my pleasure and duty when

I return to Philadelphia to see that a complete retraction is made and forwarded to each person who received a copy of the aforesaid pamphlet; and, furthermore, in the August number of the *Dental Cosmos* and the INTERNATIONAL DENTAL JOURNAL, I will see that this statement is inserted.

(Signed) JOSEPH HEAD,
*Editor of the Odontological Society.*

---

### THE HARVARD DENTAL ALUMNI ASSOCIATION.

THE Harvard Dental Alumni Association observed Monday, June 28, 1897, as "Alumni Day."

At the Harvard Dental School building, in the morning, the work of the freshman, junior, and senior classes for the past year was shown, and clinics and demonstrations given.

A large number of patients with fractured jaws were present, showing the conditions arising in the oral cavity and by plaster casts with appliances *in situ*. Likewise were shown patients with cleft palate and orthodontia conditions.

One hundred and fifty-two visitors (aside from patients) were registered and shown throughout the building, being met at the entrance by members of the reception committee.

In the surgical department was successfully performed, by Professor Thomas Fillebrown, an operation in staphylorrhaphy upon a child six years of age.

Dr. Dwight M. Clapp exhibited the X-ray apparatus, and showed, by means of a patient in the chair, its utility and practical value in dentistry.

Three entertainments for the afternoon were provided, viz.,—a barge ride through historic Cambridge and inspection of University buildings; twenty persons took this trip; nine members took the bicycle run through the park system, and thirteen enjoyed themselves at the theatre.

Five o'clock, P.M., found eighty-eight persons gathered at Young's Hotel, Boston, who participated at the twenty-sixth annual banquet of the Association.

Reports of officers were submitted, showing the Association to be in a flourishing condition. Rev. George Hodges, A.M., D.D., of Cambridge, Mass., the guest and orator of the evening, spoke upon the topic "Since the First of January," covering the events of the past six months. The growth and success of the school was described by Professor E. H. Smith, Dean of the Dental School, who said, among other things, that in eight years the school had secured forty-eight thousand dollars, that for the past year there were one hundred and thirty-two pupils in the various departments of the school, and that the incoming freshman class numbered at least thirty men. The graduating class numbered thirty-two, the largest number ever receiving the degree in a single year.

Charles W. Berry, '97, responded in a humorous vein for the class.

During the progress of the dinner the election of officers took place, and resulted as follows: President, Joseph T. Paul, '91, Boston; Vice-President, Frederick Bradley, '86, Newport, R. I.; Secretary, Waldo E. Boardman, '86, Boston; Treasurer, Harry S. Parsons, '92, Boston.

*Executive Committee.*—Waldo E. Boardman, '86, Boston; William P. Cooke, '81, Boston; Frank T. Taylor, '90, Boston.

The council is composed of the officers of the Association.

WALDO E. BOARDMAN,
*Secretary.*

BOSTON, July 7, 1897.

---

### WOMAN'S DENTAL ASSOCIATION OF THE UNITED STATES.

THE fifth annual meeting of the Woman's Dental Association of the United States was held at the office of Dr. Mary H. Stillwell, 1718 Walnut Street, Philadelphia, March 6, 1897.

The following officers were elected for the ensuing year: President, Dr. E. Davis; Vice-President, Dr. M. B. Rauch; Recording Secretary, Dr. Frances G. Crouch; Corresponding Secretary, Dr. A. S. Focht; Treasurer, Dr. C. M. Wyeth.

*Executive Committee.*—Drs. Mary H. Stillwell, Martha A. Corkill, Maria S. Lasser, Hannah Miller, and Eliza T. Yerkes.

The Vice-Presidents from representative States are Drs. Sara May Townsend, of Colorado; Edith Jewell, of Washington, D. C.; Hester Baker, of Illinois; Mary Gallop, of Massachusetts; Fannie C. Hoops, of Maryland; May Weston, of Missouri; Alice Ireland, of New York; Cora S. Little, of Nebraska; Sarah Gardiner, of Wyoming, and Jennie H. Gallop, of Rhode Island.

The membership numbers thirty-four

The June clinic was held at Dr. Davis's office on Walnut Street. Demonstration with soft gold by Dr. Davis.

Meeting adjourned to meet in the early fall.

FRANCES G. CROUCH,
*Recording Secretary.*

302 SOUTH TENTH STREET, PHILADELPHIA.

---

### RECENT PATENTS.

A LIST of recent dental patents reported for the INTERNATIONAL DENTAL JOURNAL:

No. 581,986.—Dental chair. Aaron P. Gould, Canton, Ohio. Filed September 16, 1896.

No. 582,045.—Dental plate. William S. Depew, Jamestown, New York. Filed December 30, 1896. Assigned to Milo Harris, same place.

No. 582,213.—Dental matrix. Edward B. Lodge, Cleveland, Ohio. Filed December 24, 1896.

No. 582,342.—Method of treating artificial dentures. Edward L. Chaffin, Helena, Ark. Filed August 31, 1896.

No. 582,731.—Dental articulators. Frank Fourt, Fairfield, Iowa. Filed February 5, 1896.

No. 582,796—Dental mandrel. Charles J. Peterson, Dubuque, Iowa. Filed June 25, 1896.

No. 583,307.—Dental rubber. Isaac B. Kleinert, New York, N. Y. Filed February 28, 1897.

No. 583,472.—Dental disk mandrel. Henry Heath, Jr., Brooklyn, N. Y. Filed June 10, 1896.

No. 583,565.—Dental bridge-work. Cassius M. Carr, Los Angeles, Cal. Filed April 19, 1895.

No. 583,625.—Dental hand-piece. Comegys C. Lusby and John Lusby, Philadelphia, Pa. Filed September 25, 1895.

No. 583,735.—Dental polishing disk. Carroll W. Dodge, Worcester, Mass. Filed December 6, 1895.

No. 583,848.—Dental impression cup. Robert A. Dunlap, Carrollton, Ohio. Filed April 1, 1897.

No. 584,345.—Artificial denture. Abraham L. Gilmer and Benjamin F. Gilmer, Quincy, Ill. Filed August 10, 1896.

No. 584,696.—Device for preventing stammering. Newton

Monday, Plattsburg, Mo., assignor of one-half to John F. Deberry, same place. Filed October 1, 1896.

No. 585,305.—Tooth-crown holder. James K. Burgess, Baltimore, Md. Filed October 30, 1896.

No. 585,358.—Tooth-brush. Frank D. Gould, Port Richmond, N. Y. Filed October 29, 1896.

No. 585,494.—Dental engine mallet. Clyde E. Williams, Springfield, Mo. Filed June 30, 1896.

Trade-Mark.—No. 29,987.—Tooth-brushes and other brushes. Charles Loonen, Paris, France. Filed April 10, 1897. The word "Comilo."

---

### POSTPONEMENT OF MEETING.

The Executive Committee of the American Dental Society of Europe has decided to postpone the meeting arranged for this year in London until August, 1898.

The next meeting will celebrate the Twenty-fifth Anniversary of the founding of this Society, and it is hoped that all members will participate in making it a brilliant success.

By order of the Executive Committee.

L. A. O'Brian,
*Secretary.*

15 Walpurgisstrasse, Dresden.

---

### NORTHERN OHIO DENTAL SOCIETY.

At the annual meeting, held at Put-in-Bay, June, 1897, the following officers were elected for the ensuing year:

President, L. P. Bethel, Kent; Vice-President, L. L. Barber, Toledo; Corresponding Secretary, W. T. Jackman, Cleveland; Recording Secretary, F. W. Knowlton, Akron; Treasurer, W. H. Fowler, Painesville.

F. W. Knowlton,
*Secretary.*

